# Functional dependence and the mental dimension of quality of life in Hemodialysis patients: the PROHEMO study

**DOI:** 10.1186/s12955-020-01464-3

**Published:** 2020-07-17

**Authors:** Gabriel Brayan Gutiérrez-Peredo, Márcia Tereza Silva Martins, Fernanda Albuquerque da Silva, Marcelo Barreto Lopes, Gildete Barreto Lopes, Antonio Alberto Lopes

**Affiliations:** 1 Graduate Program in Medicine and Health, Federal University of Bahia, Salvador, BA Brazil; 2 Clinic of Renal Disease and Hypertension (CLINIRIM), Salvador, BA, Brazil; 3 NEPHRON Clinic, Salvador, BA Brazil; 4 Unit of Clinical Epidemiology and Evidence Based Medicine, Professor Edgard Santos University Hospital, Federal University of Bahia, Salvador, BA, Brazil; 5 Department of Internal Medicine, Federal University of Bahia, Av. Reitor Miguel Calmon, s/n, Vale do Canela, Salvador, Bahia CEP: 40110-010 Brazil

**Keywords:** Quality of life, Depression, Mental health, Functional status, Dialysis, Activities of daily living

## Abstract

**Background:**

Functional dependence is highly prevalent in maintenance hemodialysis (MHD) settings. Also, poor health-related quality of life (HRQoL) and high levels of depressive symptoms have been reported by MHD patients. We investigated associations between functional status and mental aspects of quality of life in Brazilian MHD patients.

**Methods:**

Cross sectional study of 235 patients enrolled in two of the four participating MHD clinics of the Prospective Study of the Prognosis of Chronic Hemodialysis Patients (PROHEMO) in Salvador, BA, Brazil. Data were collected from September 2016 to August 2017. The Katz’s questionnaire was used for basic activities of daily living (ADL) and the Lawton-Brody’s questionnaire for instrumental activities of daily living (IADL). ADL and IADL scores were combined to create 3 functional status groups: highly dependent (*n* = 47), moderately dependent (*n* = 109) and independent (*n* = 82). The validated Brazilian version of the Kidney Disease Quality of Life Short Form (KDQOL-SF) was used for scores of two distinct HRQoL measures, i.e., the mental component summary (MCS) and the 5-item mental health inventory (MHI-5). We used linear regression to estimate differences in scores with adjustment for possible confounders: months of dialysis, age, gender, other sociodemographic variables, body mass index, type of vascular access, dialysis dose by Kt/V, laboratory variables (albumin, blood hemoglobin, calcium, phosphorus, urea, creatinine and parathyroid hormone) and nine comorbid conditions.

**Results:**

Mean age was 51.2 ± 12.4 yr (median age = 51.0 yr), 59.1% were male, 93.2% were non-White. The prevalence of self-reported functional status differed by age: 54.4% for age < 45 yr, 67.8% for age 45–60 yr and 73.9% for age ≥ 60 yr. Using functionally independent as reference, lower scores were observed for highly dependent patients in MCS (difference: -4.69, 95% CI: -8.09, -0.29) and MHI-5 (difference: -5.97, 95% CI: -8.09, -1.29) patients. These differences changed slightly with extensive adjustments for covariates.

**Conclusions:**

Our results call attention to a high prevalence of functional dependence in younger and older MHD patients. The results suggest that the lower self-reported mental quality of life in functionally dependent MHD patients cannot be explained by differences in age and comorbidities.

## Background

Functional status is related to the individual’s ability to perform basic activities of daily living (ADL) and self-care instrumental activities of daily living (IADL) [1, 2]. ADL includes daily activities, such as eating, dressing, bathing, using the toilet and moving from one position to another. IADL includes more complex and refined activities that most people learn during their teenage years or early adulthood, such as grocery shopping, meal preparation and household chores. Reduction in functional status will lead patients to rely on others to perform their ADLs and IADLs.

Functional dependence is a problem expected to arise at an elder age, but may become apparent earlier in the lives of patients with chronic disease, as is the case of chronic kidney disease, including end-stage renal disease (ESRD) patients undergoing maintenance hemodialysis (MHD) [3–7]. Data from the Dialysis Outcomes and Practice Patterns Study (DOPPS), for example, show a high prevalence of functional dependence among younger and older ESRD patients undergoing MHD in the United States, Canada, European countries, Japan, New Zealand and Australia [7]. A high prevalence of comorbid conditions is also observed in MHD patients, particularly among those who report functional dependence [8, 9]. ESRD patients undergoing MHD also report, in general, poor health-related quality of life (HRQoL) and high levels of depressive symptoms [10, 11]. Moreover, there are data to indicate that functional status is an independent predictor of mortality in MHD patients [7, 12]. Studies are needed to investigate associations between self-reported functional status and psychological well-being in MHD patients and possible influences of potential confounders, such as, age and comorbidities in the association.

The main objectives of the present study in patients undergoing MHD in a large Brazilian city was to estimate the prevalence of functional dependence and investigate associations of functional status with mental aspects of quality of life. We assessed possible influences of age, and comorbidities in the comparisons of the HRQoL measures between patients with different levels of functional status.

## Methods

### Study design and sample

The study was a cross-sectional analysis of data of the Prospective Study of the Prognosis of Chronic Hemodialysis Patients (PROHEMO) [13–15], developed at satellite dialysis units in the city of Salvador (Bahia), Brazil. This Northeastern Brazilian town has a population of approximately three million people, 80% of which are Black or Mixed-race [16].

Data collection was initiated in 2016 when the questionnaires for functional status started to be applied to PROHEMO participants. The analysis was restricted to two (here identified as Clinic A and Clinic B) of the original four participating dialysis clinics of PROHEMO [14]. Approximately 19% of the patients were undergoing hemodialysis for less than 1 year, 50% for less than 4 years and 80% for less than 8 years at the time of data collection. In Clinic A, all 211 adult patients undergoing treatment from September 2016 to May 2017 were invited to participate. Among the 191 patients who agreed to participate, 179 were interviewed and 12 were not able to respond to the questionnaires because of cognitive impairment. In Clinic B, 70 patients were randomly selected from a population of 311 adult patients undergoing treatment in June 2017. Among 70 patients in Clinic B, 64 were interviewed from July 2017 to August 2017, four did not agree to participate and two were not able to respond to the questionnaires because of cognitive impairment.

From the sample of 243 patients, 235 (173 from Clinic A and 62 from Clinic B) had data for both functional status and HRQoL measures. These 235 patients constitute the sample of the present analysis. The Institutional Review Board of the Medical School of the Federal University of Bahia approved the research protocol of the present study and all patients provided informed consent to participate.

### Data collection and definitions

The collection of demographic, laboratory and clinical data began as soon as the patients provided informed consent to participate. The data were provided by the patient and supplemented with information from the attending nephrologist or extracted from medical records. The patient was classified by race as White or non-White by pre-defined criteria [17]. To determine economic class (A - E) we used the classification system of the Brazilian Institute of Market Research which is primarily based on possession of consumer goods [18]. We classified patients belonging to classes D and E as poor or very poor. We used the laboratory results closest to the collection of baseline clinical data and patient’s self-reported data on functional status and HRQoL. Blood samples for laboratory tests were collected before the dialysis session.

### Predictors and outcomes

The predictor variable was functional status. The outcome variables were the scores of the mental component summary (MCS) and the mental health inventory (MHI-5). We used the ADL Katz’s questionnaire [19] and the IADL Lawton-Brody’s questionnaire [20, 21] to assess functional status. Katz’s questionnaire assesses the level of ability of the patient to perform 5 ADL tasks (Table 1). Interviewers asked patients if they were able to perform the following tasks, without assistance: eating, getting dressed, bathing, using the toilet and/or transferring from bed to chair. The options of responses for each ADL item were “yes” or “no”. The Lawton-Brody’s questionnaire was used to evaluate ability to 8 IADL tasks (Table 1). Patients were asked to choose the best answer to describe their ability to perform each of the following activities: using a telephone, getting places beyond walking distance, grocery shopping, preparing meals, doing housework or handyman work, doing laundry, taking medication or managing money. The options of responses for each IADL item were “need no help,” “need some help,” or “unable to do at all”. For the ADL, a “yes” response was scored as 1 and a “no” response was scored as 0.25 considering that was not possible to distinguish if the patient performed the task with some help or if he or she was unable to perform the task at all. For the IADL responses, “need no help” were scored 1; “need some help,” scored 0.5; and “unable to do at all” scored 0. Adequate to high internal consistency has been demonstrated earlier for the Katz’s ADL [22, 23] and the Lawton-Brody’s IADL instruments [20, 21]. In the present study, the Cronbach’s alpha for the 5 ADL questions was 0.94 and for the 8 items of the Lawton-Brody questions was 0.87.

**Table 1:** Distribution of responses for the tasks of the Activities of Daily Living (ADL) and the Instrumental Activities of Daily Living (IADL) in the Study Sample

**Activities of Daily Living (ADL)**			
	Ability to perform without help: number (%)		
Tasks	Yes (score = 1)	No (score = 0.25)	
Eating	230 (97.9)	5 (2.1)	
Getting dressed	228 (97.0)	7 (3.0)	
Bathing	226 (96.2)	9 (3.8)	
Using the toilet	224 (95.3)	11 (4.7)	
Transferring from bed to chair	225 (95.7)	10 (4.3)	
**Instrumental Activities of Daily Living (IADL)**			
	Ability to perform without or with some help: number (%)		
Tasks	Do not need help (score = 1)	Need some help (Score = 0.5)	Unable to do at all (Score = 0)
Using the telephone	218 (92.8)	16 (6.8)	1 (0.4)
Getting places beyond walking distance	139 (59.1)	81 (34.5)	15 (6.4)
Grocery shopping	110 (46.8)	110 (46.8)	15 (6.4)
Preparing meals	178 (75.7)	51 (21.7)	6 (2.6)
Doing housework or handyman work	143 (60.9)	81 (34.5)	11 (4.7)
Doing laundry	163 (69.4)	68 (28.9)	4 (1.7)
Taking medications	206 (87.7)	29 (12.3)	0 (0)
Managing money	207 (88.1)	27 (11.5)	1 (0.43)

The ADL and IADL scales were combined to create an expanded functional status scale. This approach was used to provide a more comprehensive evaluation of the functional status of the patients and it is supported by research on the psychometric properties [24–26]. The results of an analysis of a nationally representative survey of 25,470 adults using Guttman and item response theory scaling methods indicated that the expanded ADL/IADL scale was less biased by age and more useful than the ADL to assess functional status in individuals of different ages [24]. The predictive validity of the expanded scale for health outcomes was demonstrated in the international DOPPS that showed a strong association of greater functional dependence with mortality, dialysis therapy withdrawal, and hospitalization [7].

We determined the overall functional status score by summing up the scores of the 13 items of the expanded functional status scale. The functional status scores could range from 1.25 to 13. To define the categories of functional status, we took into account the classification described in the 2016 DOPPS publication, which was based on logistic regression proportional odds model [7]. In DOPPS the functional status score was categorized into four groups: < 8, 8 - < 11, 11 - < 13, and 13. Similar to DOPPS, patients with overall score of 13 were defined as functionally independent and corresponded to those who reported no need for assistance to execute all 13 functional status tasks. The range of scores used to define the moderately dependent group (score range: ≥11 - < 13) were also the same used in DOPPS. Because the percentage of patients with overall score < 8 was small (4%), we combined the groups with scores 8- < 11 and < 8 into a single category (< 11) to define the highly dependent group.

We used the validated Brazilian version of the Kidney Disease Quality of Life Short Form (KDQOL-SF) [27] to assess aspects of the mental dimension of quality of life. The KDQOL-SF is comprised of kidney disease-targeted items and generic items from the Medical Outcomes Study 36-Item Short-Form Health Survey (SF-36) [28]. The patient responses to the questions from the generic core of KDQOL-SF were used to calculate MCS and MHI-5 scores. MCS scores were calculated by using the scoring algorithm proposed by Ware et al., which takes into account factor score coefficients for the all SF-36 domains [29]. According to the proposed algorithm for the MCS score, the coefficients are negative for physical functioning, role-physical, bodily pain and general health. The positive coefficients for MCS scores are those for vitality, social functioning, role-emotion and mental health (emotional well-being). The scores of each generic measure used for the MCS may vary from 0 to 100. However, because of the weighting used, the range of the MCS score is expected to be narrower than the ranges of the SF-36 scales used to determine the MCS score. In the present study the minimum MCS score was 18.9 and the maximum MCS score was 70.1. Higher MCS scores represent better HRQoL.

To determine scores of the MHI-5, interviewers asked patients to indicate how much of their time over the previous 4 weeks they had felt “so down in the dumps that nothing could cheer you up,” “downhearted and blue,” “very nervous,” “calm and peaceful,” and “a happy person.” Possible responses to these five questions were “All of the time,” “Most of the time,” “A good bit of the time,” “Some of the time,” “A little of the time,” or “None of the time”. The scores of the MHI-5 may range from 0 to 100. The minimum and maximum values of the MHI-5 were 8.0 and 100, respectively. Similar to the MCS score, higher MHI-5 scores also represent better HRQoL. The recommended cutoff of 52 for MHI-5 was used to categorize the patients into two groups with scores ≤52 representing worse mental quality of life [30].

### Statistical methods

We used mean ± standard deviation to describe characteristics consistent with normal distribution and median [interquartile range] to describe variables that were not consistent with a normal distribution. Percentages were used to describe frequencies of categorical attributes. A standardized effect size measure was used to assess the magnitude of the difference in scores. The effect size measure was determined by using as numerator the mean difference in scores between the two groups and as denominator the pooled standard deviation of the scores [31]. A value ≤0.20 was interpreted as small effect size, 0.50 as moderate effect size and ≥ 0.80 as large effect size.

Logistic regression and linear regression models were used to estimate associations with adjustment for potential effects of covariates. The inclusion of covariates in the multivariable models was based on characteristics shown to be associated with functional status [7, 12] and poor HRQoL in hemodialysis patients [10], independent of *p* value or statistical significance [32, 33]. The linear and logistic regression models had similar structures regarding the inclusion of covariates: Adjusted-1 model included months on dialysis and age; Adjusted-2 model included months on dialysis, age, and the other sociodemographic variables, i.e., sex, race, marital status (married vs not married), education (<high school vs ≥ high school), living status (living with family vs not living with family), economic class (poor/very poor vs higher level); Adjusted-3 model included all covariates listed in Table 2, except hypertension that was omitted because of collinearity. Economic class was the only covariate with missing values. Missing value for this covariate was present for 6 of the 235 patients (2.6%). To handle missing data, we performed multiple imputation with 20 repetitions using logistic regression with economic class as the outcome variable (code as 1 for poor or very poor and 0 for higher levels) and the set of sociodemographic variables shown in Table 2 as the independent variables. A sensitivity analysis with the 229 patients with complete data was also performed for comparison of the results. We used 95% confidence interval (95% CI) to describe the precision of the estimates. The STATA version 15.1 for Mac was used for the statistical analysis.

**Table 2:** Patient characteristics by functional status

	Functional Status		
Sociodemographics	Highly Dependent ***n*** = 44	Moderately Dependent ***n*** = 109	Functionally Independent ***n*** = 82
Age in years, mean ± SD	57.5 ± 10.3	50.7 ± 12.1	48.5 ± 12.9
Male (%)	56.8	53.2	68.3
Non-White (%)	84.1	95.4	95.1
Married (%)	54.6	42.1	50.0
<High school (%)	47.7	50.5	37.8
Live with family (%)	90.9	91.7	86.6
Poor or very poor (%)	16.3	17.8	17.7
Months on dialysis, median [IQR]	37.8 [12.0, 76.8]	48.8 [20.0, 89.4]	51.0 [19.4, 83.5]
Body mass index, mean ± SD	24.6 ± 4.2	23.6 ± 4.4	24.3 ± 4.0
Vascular access by catheter (%)	40.9	18.4	13.4
Kt/V, mean ± SD	1.47 ± 0.33	1.48 ± 0.40	1.37 ± 0.36
**Laboratory exams**			
Albumin in g/dL, mean ± SD	4.04 ± 0.62	4.09 ± 0.83	4.03 ± 0.55
Hemoglobin in g/dL, mean ± SD	10.4 ± 2.00	10.7 ± 1.99	10.7 ± 1.77
Calcium in mg/dL, mean ± SD	9.31 ± 0.64	9.25 ± 0.87	9.30 ± 0.60
Phosphorus in mg/dL, mean ± SD	5.26 ± 1.20	5.10 ± 1.34	5.53 ± 1.26
Urea, mean ± SD	150 ± 33.7	150 ± 39.1	161 ± 36.3
Creatinine in mg/dL, mean ± SD	9.16 ± 3.01	9.95 ± 3.68	11.38 ± 3.44
Intact PTH in pg/mL, median [IQR]	177 [101, 476]	235 [106, 598]	339 [188, 735]
**Comorbid conditions (%)**			
Hypertension	93.2	96.3	95.1
Diabetes	56.8	31.2	12.2
Congestive heart failure	22.7	13.8	11.0
Coronary artery disease	15.9	4.6	3.6
Cerebrovascular disease	13.6	2.7	7.3
Peripheral vascular disease	9.09	2.75	4.88
Cancer	4.55	0.00	1.22
COPD/Asthma	2.27	3.67	1.22
Chronic Liver Disease	11.4	3.67	6.10

*Abbreviations*: *IQR* Interquartile range, *PTH* Parathyroid hormone, *COPD* Chronic obstructive pulmonary disease

## Results

Our sample consisted of 235 MHD patients with a mean age of 51.2 ± 12.4 yr (median age = 51.0 yr), 59% were male and 93% were non-White. The distribution of patient’s characteristics did not differ markedly between Clinic A and Clinic B. The most common cause of ESRD was hypertensive nephropathy (26%), followed by diabetic nephropathy (25%) and glomerulonephritis (18%).

The distribution of ADL and IADL scores in the study sample of 235 patients is displayed in Table 1. The patients reported more often to be able to perform ADL than IADL tasks without help. Less than half (47%) of the patients reported to be able to shop for grocery without help. Approximately 40% of the patients needed some help or were unable to go to places beyond walking distance or to do housework/handyman work.

Table 2 describes patient characteristics by the three functional status groups. There were 44 patients in the highly dependent group (18.7%), 109 in the moderately independent group (46.4%) and 82 in the functionally independent group (34.9%). Compared to functionally independent patients, those reporting functional dependence had a higher mean age, lower concentrations of parathyroid hormone (PTH) and serum creatinine, higher proportion of hemodialysis by catheter and higher prevalence of comorbidities (except hypertension).

Table 3 shows unadjusted and adjusted associations of selected characteristics with the prevalence of self-reported moderate to greater functional dependence as compared to functional independence. The prevalence of self-reported functional dependence was 54.4% for age < 45 yr, 67.8% for age 45 to 60 yr and 73.9% for age ≥ 60 yr. The strength of the association between age and functional status was reduced with adjustments for comorbidities and laboratory variables. The prevalence of functional dependence was higher for diabetics (85%) than for non-diabetics (56.6%) and for patients undergoing dialysis by catheter (77.6%) than by arteriovenous fistulae (61.8%). The strength of the associations between functional status with both diabetes and dialysis by catheter were not reduced with adjustments for covariates. Higher prevalence of self-reported functional dependence was also observed for patients with serum PTH < 150 pg/mL (89.2% for PTH < 150 pg/mL vs 40.9% for PTH > 300 pg/mL) and for patients with serum creatinine ≤9 mg/dL (81.3% for creatinine ≤9 mg/dL vs 54.9% for creatinine > 9 mg/dL). The associations of low serum PTH and creatinine concentrations with higher likelihood of functional dependence were observed both in models with minimal and extensive adjustments for covariates.

**Table 3:** Prevalence of functional dependence and associations of functional status with patient’s characteristics

			Odds Ratio and 95% Confidence Interval			
Functional Status	N	% Dependent	Unadjusted	Adjusted-1	Adjusted-2	Adjusted-3
**Age** (years)						
< 45	79	54.4	Ref = 1	Ref = 1	Ref = 1	Ref = 1
45–60	87	67.8	1.76 (0.94,3.32)	1.76 (0.94, 3.32)	1.67 (0.86, 3.24)	1.09 (0.49, 2.42)
≥ 60	69	73.9	2.37 (1.18, 4.76)	2.38 (1.18, 4.76)	2.45 (1.15, 5.18)	1.34 (0.54, 3.35)
**Diabetes**						
No	166	56.6	Ref = 1	Ref = 1	Ref = 1	Ref = 1
Yes	69	85.5	4.52 (2.16, 9.45)	4.59 (2.09, 10.1)	4.74 (2.10, 10.7)	8.15 (2.94, 22.6)
**Vascular Access for Hemodialysis**						
AVF	166	61.8	Ref = 1	Ref = 1	Ref = 1	Ref = 1
Catheter	69	77.6	2.13 (1.02, 4.44)	2.29 (1.08, 4.87)	2.08 (0.96, 4.52)	2.41 (0.91, 6.35)
**PTH (**pg/mL**)**						
> 300	149	40.9	Ref = 1	Ref = 1	Ref = 1	Ref = 1
150–300	123	63.4	1.20 (2.12, 10.5)	1.20 (0.63, 2.30)	1.32 (0.67, 2.60)	0.90 (0.37, 2.19)
< 150	83	89.2	4.72 (2.12, 10.5)	4.81 (2.11, 11.0)	5.37 (2.27, 12.7)	3.80 (1.23, 11.7)
**Serum Creatinine (**mg/dL**)**						
> 9	144	81.3	Ref = 1	Ref = 1	Ref = 1	Ref = 1
≤ 9	91	54.9	3.58 (1.93, 6.66)	3.62 (1.89, 6.95)	3.09 (1.96, 6.54)	3.66 (1.48, 9.07)

Adjusted-1: Includes months on dialysis and age; Adjusted-2: Includes months on dialysis, age and other sociodemographics; Adjusted-3: Includes vascular access by catheter, comorbidities, laboratory variables, BMI, plus variables in Adjusted-2

*AVF* Arteriovenous fistulae

Table 4 shows the results from the regression analysis of the association between functional status and scores of HRQoL measures (MCS and MHI-5). Compared with functionally independent, the MCS score was lower by approximately 5 points for the moderately dependent (difference: -4.56, 95% CI: -8.30, -1.60; effect size: 0.42). Similar difference in MCS score was observed in the comparison between highly dependent and functionally independent (difference: -4.65, 95% CI: -9.00, -0.31; effect size: 0.39). The differences in MCS scores by functional status changed slightly with adjustments for months on dialysis, treatment variables, laboratory exams and comorbid conditions.

**Table 4:** Associations between the mental component summary and the mental health inventory scores with functional status

**Mental Component Summary (MCS)**						
	**Score**	**Effect**	**Difference in Mean Score (95% Confidence Interval)**			
**Functional Status**	**mean ± SD**	**Size**	**Unadjusted**	**Adjusted-1**	**Adjusted-2**	**Adjusted-3**
Independent	52.7 ± 10.7	Ref	Ref	Ref	Ref	Ref
Moderately Dependent	48.2 ± 11.1	0.42	-4.56 (-8.3, -1.6)	-5.01 (-8.34, -1.68)	-4.66 (-8.06, -1.27)	-4.46 (-8.14, -0.79)
Highly Dependent	48.1 ± 13.2	0.39	-4.65 (-9.00, -0.31)	-6.07 (-10.5, -1.65)	-5.97 (-10. 5, -1.46)	-5.25 -10.3, -0.16)
**Mental Health Inventory (MHI-5)**						
	**Score**	**Effect**	**Difference in Mean Score (95% Confidence Interval)**			
**Functional Status**	**mean ± SD**	**Size**	**Unadjusted**	**Adjusted-1**	**Adjusted-2**	**Adjusted-3**
Independent	81.4 ± 21.1	Ref	Ref	Ref	Ref	Ref
Moderately Dependent	73.9 ± 21.5	0.35	-7.42 (-13.6, -1.21)	-8.30 (-14.4, -2.19)	-7.29 (-13.5, -1.10)	-6.72 (-13.3, -0.13)
Highly Dependent	67.5 ± 22.6	0.63	-13.8 (-21.8, -5.88)	-17.0 (-25.1, -8.97)	-16.4 (-24.6, -8.28)	-14.5 (-23.6, -5.37)

Ref = Reference group used for comparisons

Adjusted-1: Includes months on dialysis and age; Adjusted-2: Includes months on dialysis, age and other sociodemographics; Adjusted-3: Includes vascular access by catheter, comorbidities, laboratory variables, BMI, plus variables in Adjusted-2

Compared with the MCS scores, a steadier reduction in the MHI-5 scores from the functionally independent to the highly dependent groups was observed (Table 4). Using functionally independent as reference, it was observed that the MHI-5 score was lower by approximately 7 points for the moderately dependent (difference: -7.42, 95% CI: -13.6, -1.21; effect size: 0.35) and by almost 14 points for the highly dependent (difference: -13.8, 95% CI: -21.8, -5.88; effect size: 0.63). As observed for MCS, the differences in the MHI-5 by functional status did not change markedly with extensive adjustments for covariates.

Similar results of the association between functional status with MCS and MHI-5 were observed in a sensitivity analysis restricted to a sample of 229 patients without missing data. Similar results were also observed using the complete sample of 235 patients with indicator variables, instead of imputation, to account for missing data on economic class. These results are not shown in the Table.

In an analysis using data of the whole sample, the MCS scores stratified for the age groups < 45 yr, 45–60 yr and > 60 yr were, respectively, 47.0 ± 12.2, 51.9 ± 9.82 and 50.5 ± 12.1. The corresponding MHI-5 scores by these age categories were 70.7 ± 24.4, 77.7 ± 19.8 and 77.6 ± 21.4. In linear models with extensive adjustments for the covariates listed in Table 2 and with age < 45 yr as reference the MCS score was higher by approximately 6.4 points for age 45–60 yr (difference: 6.4, 95% CI: 2.3, 10.4) and 3.9 points for age > 60 yr (difference: 3.9, 95% CI: -0.4, 8.3). For MHI-5, the score was higher by approximately 8.6 points for age 45–60 yr (difference: 8.6, 95% CI: 1.2, 16.1) and 6 points for ages > 60 yr (difference: 6.0, 95% CI: -2.1, 14.0). These results are not shown in the Table.

The prevalence of MHI-5 ≤ 52 was 8.5% (7/82) for functionally independent, 20.2% (22/109) for moderately dependent and 25% (11/44) for highly dependent patients. The prevalence of MHI-5 ≤ 52 points was approximately 2.5 times higher (prevalence ratio 2.53, 95% CI: 1.17, 5.46) in patients who reported any degree of functional dependence than in those functionally independent. The results of the multivariate logistic regression show that the direction of the association between functional status and MHI-5 ≤ 52 did not change and the strength of association was not reduced with extensive adjustments for covariates. These results are not shown in Table 4.

We also evaluated the data to estimate differences in MCS and MHI-5 scores by ADL (5 versus lower score) and the IADL tasks (need help versus not need help). In the comparisons between ADL task groups, larger difference was observed for MHI-5 (70.7 ± 22.8 for ADL score < 5 and 75.6 ± 22.0 for ADL score = 5, effect size: 0.22), than for MCS (48.4 ± 13.0 for ADL score < 5 and 49.8 ± 11.5 for ADL score = 5, effect size: 0.11). In the comparisons between IADL task groups, larger differences were also observed for MHI-5 than for MCS. For the task “go to places beyond walking distance”, the MCS score were 47.8 ± 12.4 in patients who need help and 51.0 ± 10.7 in patients who did not need help, with effect size of 0.28. The MHI-5 scores between patients who need and do not need help for the task “walking distance” were, respectively, 69.2 ± 23.1 and 79.6 ± 20.3, with effect size of 0.48. For the task “be able to shop for grocery,” the MCS score were 47.9 ± 11.5 in patients who need help and 51.8 ± 11.3 in patients who did not need help, with effect size of 0.34. The MHI-5 scores between patients who need and do not need help for the task “shop for groceries” were, respectively, 71.3 ± 21.7 and 79.9 ± 21.6, with effect size of 0.40. For the task “doing housework or handyman work”, the MCS score were 47.4 ± 11.9 in patients who need help and 51.2 ± 11.1 in patients who did not need help, with effect size of 0.33. The MHI-5 scores between patients who need help and those who do not need help for the task “housework/handyman work” were, respectively, 69.9 ± 22.5 and 78.8 ± 21.1, with effect size of 0.41. The results of the comparisons of MCS and MHI-5 scores by ADL and IADL tasks are not shown in the Table.

## Discussion

This study shows a very high prevalence of patient-reported functional dependence in a sample of Brazilian ESRD patients undergoing MHD; more than half of the participants reported functional dependence. In general, the patients reported more difficulty in performing IADL than ADL tasks. The prevalence of self-reported functional dependence in MHD patients observed in our study was similar to that reported in a DOPPS analysis of MHD patients treated in 12 countries [7] and, apparently, was much higher than in prior reports for older adults in the general population [34]. The results indicate that self-reported functional status is strongly associated with poorer mental quality of life in MHD patients. The average MCS and MHI-5 scores were much lower for patients who reported to be moderately or highly functionally dependent than the functionally independent patients. In the comparisons using the overall functional status scores, the reduction in the scores of the mental components of quality of life from the functionally independent (overall score 13) to the highly dependent group (overall score < 11) was more remarkable for MHI-5 than for MCS, following a monotonic pattern. The larger differences in the MHI-5 than in the MCS scores were also observed both for the comparisons between ADL task groups and between IADL task groups.

Functionally dependent MHD patients were, in general, older and presented higher prevalence of comorbidities. However, the associations between functional status and the mental components of quality of life were not attenuated after adjustment for age and preexisting comorbidities. This finding may be explained, at least partially, by effects of age on functional status and the mental components of quality of life. Whereas the prevalence of self-reported functional dependence was higher for the older patients, the scores of MCS and MHI-5 was higher for the older than for the younger patients. Similar results on the associations between age and HRQoL scores in MHD patients were observed in the international DOPPS. In a sample of 9526 MHD patients enrolled in DOPPS, it was observed that, differently from the Physical Component Summary (PCS), older age was not associated with lower MCS score [10]. Moreover, no association was seen between age and depression symptoms, assessed by the Center for Epidemiological Studies Depression Index (CES-D), in another DOPPS investigation [11]. These findings are consistent with the claim that patient experiences, expectations and perceptions regarding health could influence their HRQoL, independent of age and health status [35, 36].

Patients who referred to be functionally dependent were more often undergoing hemodialysis by catheter and had lower serum concentrations of creatinine and PTH. The higher prevalence of hemodialysis by central venous catheter (compared with arteriovenous fistulae) in functionally dependent patients is consistent with previous observations [37, 38]. It has been suggested that if the patient has a poor functional status or limited life expectancy, the placement of an arteriovenous fistulae may be postponed until after the start of hemodialysis [38].

The association of low PTH and low serum creatinine with higher likelihood of functional dependence in the present study could not be explained by effects of age and comorbidities. Prior evidence suggests that lower concentration of PTH observed in patients referring functional dependence might be due to adynamic bone disease, a prevalent cause of pain and bone fractures in MHD patients [39]. The lower serum creatinine in patients reporting functional dependence is consistent with possible role of reduced muscle mass and poor nutritional status in the functional status of MHD patients [40–42]. These findings may suggest an influence of protein energy wasting and inflammation in sicker MHD patients [43].

While the present study offers insights into the association between functional status and the mental quality of life of MHD patients, methodological limitations cannot be ignored. Because the study is cross-sectional, causal inference cannot be reached and directional ambiguity in the association is possible. Although a stronger monotonic association of functional status with MHI-5 than with MCS scores was observed, it is not possible to conclude that the MHI-5 scale is more sensitive than the MCS scale to changes in the functional status of MHD patients considering that cross-sectional differences in HRQoL score may not equate to longitudinal changes [36]. Our results suggest poor mental quality of life in MHD patients who report functional dependence, but they do not permit to identify what disorder, such as, depressive symptoms and anxiety play a major role in the association of functional status with MCS and MHI-5. Another limitation is to determine if the differences in HRQoL scores are clinically significant considering that it depends on several factors, including the criteria to define what constitute clinically important difference in scores, the patient’s perspective, and if the HRQoL was assessed using data collected at single time point in a cross-sectional study or using repeated measures in an observational cohort study or in a randomized clinical trial [44–46]. Differences of at least 3–5 points in the SF-36 scales have been proposed as a clinically relevant or minimum clinically important difference (MCID) [47, 48]. However, this proposal for the use of mean difference to define clinical relevance has been criticized and the use of a standardized effect size measures has been favored by some experts [49, 50]. Samsa et al. advocate a threshold of 0.20 in standardized effect size as clinically relevant [50]. The observed differences in the scores of MCS and MHI-5 between functional status categories in the present study exceeded the threshold proposed for MCID, both for mean difference and effect size, but we should be cautious to conclude that the observed differences are relevant in the context of clinical practice considering the observational, cross-sectional, design of the study.

Generalizability of findings from the present study to other hemodialysis populations have to be pondered, due to unique cultural and demographic characteristics of our study population, composed of more than 90% non-White (mostly of African descent) participants. Additionally, the mean age (< 60 yr) in our study sample is lower than that of studies with MHD patients developed outside Brazil, especially in developed countries.

## Conclusions

This study calls attention to a very high prevalence of self-reported functional dependence in younger and older MHD patients. The results suggest that functionally dependent patients have poorer mental quality of life than functionally independent patients. According to the results, the poorer mental quality of life associated with functional dependence cannot be explained by age and the presence of prevalent comorbid conditions in MHD patients.

## Data Availability

Data is deposited in the repository of the Federal University of Bahia, Salvador, BA, Brazil.

## Abbreviations

ADL: Activities of daily living
CI: Confidence interval
COPD: Chronic obstructive pulmonary disease
CES-D: Center for Epidemiological Studies Depression Index
DOPPS: Dialysis Outcomes and Practice Patterns Study
ESRD: End-stage renal disease
HIV/AIDS: Human immunodeficiency virus/Acquired immune deficiency syndrome
HRQoL: Health-related quality of life
IADL: Instrumental activities of daily living
IQR: Interquartile range
KDQOL-SF: Kidney Disease Quality of Life Short Form
MCID: Minimum clinically important difference
MCS: Mental component summary
MHD: Maintenance Hemodialysis
MHI-5: 5-Item Mental health inventory
PCS: Physical Component Summary
PROHEMO: Prospective Study of the Prognosis of Chronic Hemodialysis Patients
PTH: Parathyroid hormone
